# Supplementary material for: Antigen-Dependent Adjuvanticity of Poly(lactic-co-glycolic acid)-polyethylene Glycol 25% Nanoparticles for Enhanced Vaccine Efficacy
Source: Vaccines (Basel). 2025 Mar 16;13(3):317. doi: 10.3390/vaccines13030317 (PMC11946064; doi:10.3390/vaccines13030317)
Supplement: Supplementary file 1 [file vaccines-13-00317-s001.zip › vaccines-3454176-supplementary.pdf]

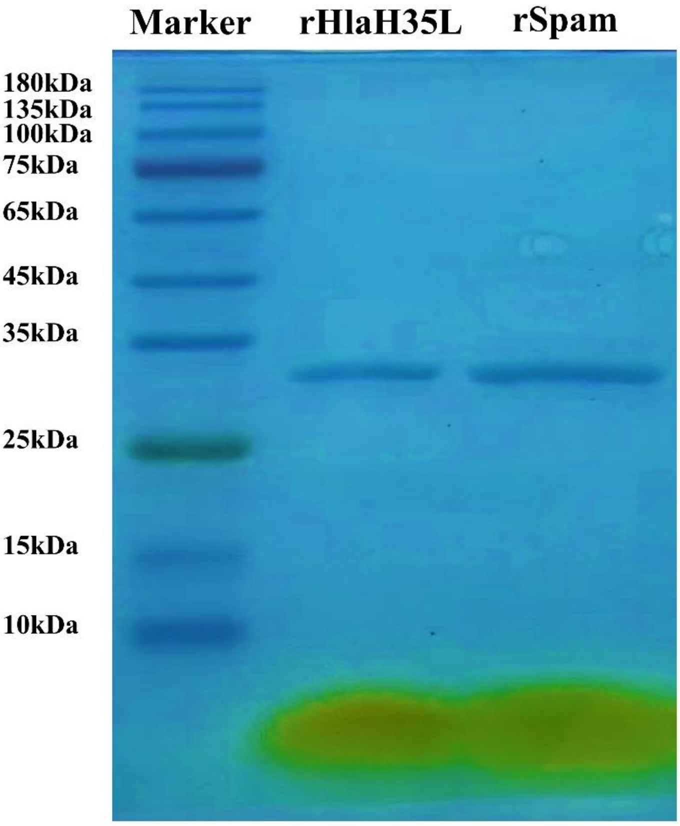

Figure S1.two antigens were analyzed by SDS-PAGE. Lane 1, marker, Lane 2, rHlaH35L. Lane 3, rSpam. Expected molecular weight of rHlaH35L is ~ 33 kDa (293 aa). Expected molecular weight of rSpam is ~ 32 kDa (291 aa).

Table 1. Hydrodynamic diameter of nanoparticles and nano-vaccines.

	25% NPs(nm)	25% NPs-rHlaH35L(nm)	25% NPs-rSpam(nm)
Batch 1	174.2 ± 4.44	200.1 ± 7.92	202.1 ± 6.77
Batch 2	169.1 ± 6.21	187.2 ± 6.21	191.7 ± 4.12
Batch 3	177.5 ± 8.12	193.1 ± 7.89	196.2 ± 7.23
Batch 4	172.9 ± 6.95	189.9 ± 9.53	201.5 ± 8.834
Batch 5	170.7 ± 5.12	196.2 ± 5.72	205.6 ± 8.12
Batch 6	173.6 ± 4.92	192.0 ± 4.22	203.9 ± 6.54

Table 2. Zeta potential of nanoparticles and nano-vaccines.

	25% NPs(mV)	25% NPs-rHlaH35L(mV)	25% NPs-rSpam(mV)
Batch 1	−17.917 ± 1.107	−8.974 ± 0.956	−22.074 ± 1.898
Batch 2	−16.923 ± 1.037	−8.265 ± 0.385	−23.183 ± 2.462
Batch 3	−18.211 ± 2.000	−7.992 ± 0.826	−20.362 ± 3.018
Batch 4	−17.892 ± 0.925	−7.362 ± 1.036	−21.684 ± 2.872
Batch 5	−16.275 ± 1.673	−9.379 ± 1.623	−22.569 ± 1.992
Batch 6	−18.209 ± 2.836	−9.002 ± 1.362	−21.473 ± 1.274

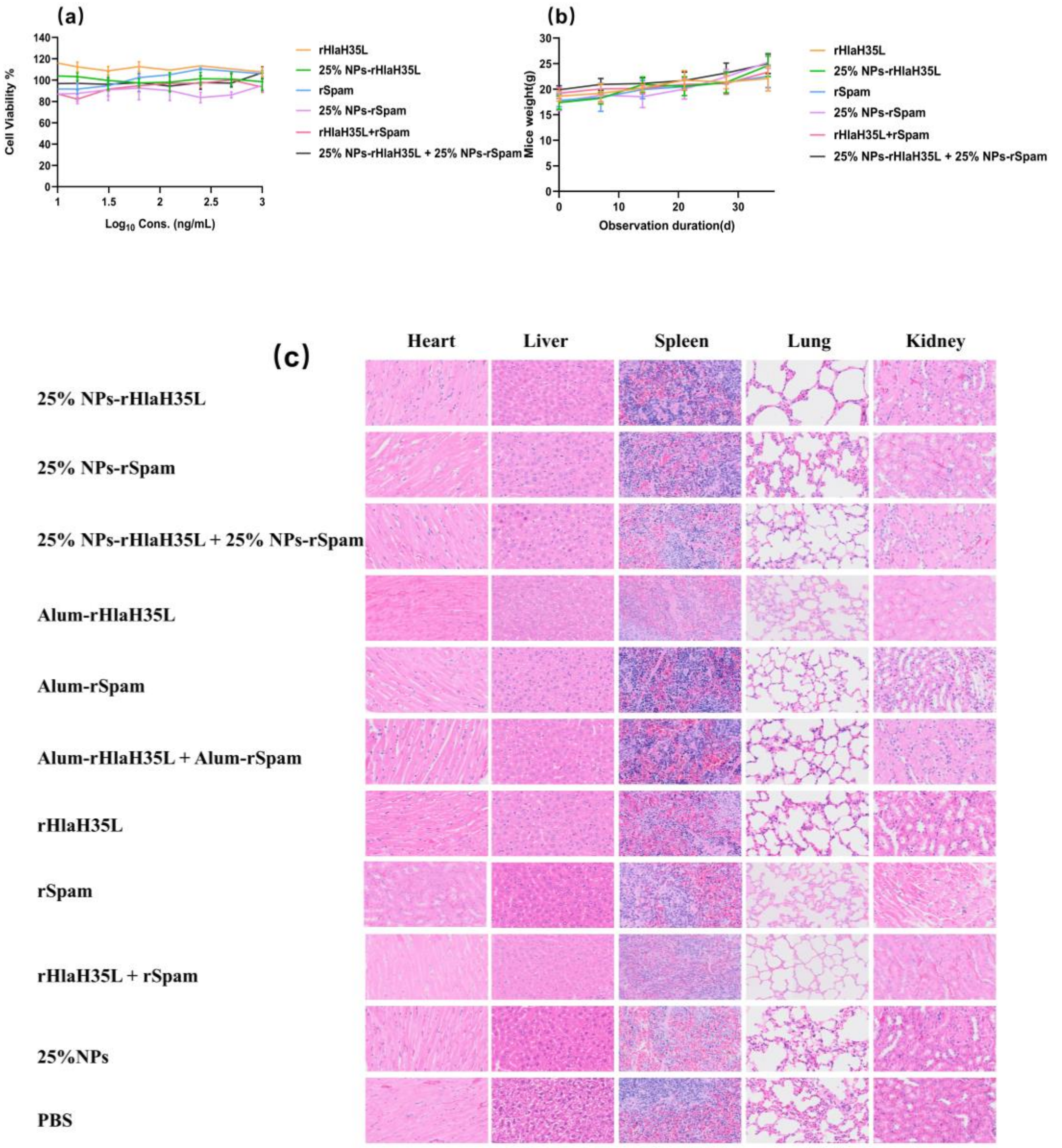

Figure S2. Biocompatibility evaluation of nano-vaccines. (a) Cell viabilities. (b)Body weight change of the mice and (c)H&E staining of major organs of the mice

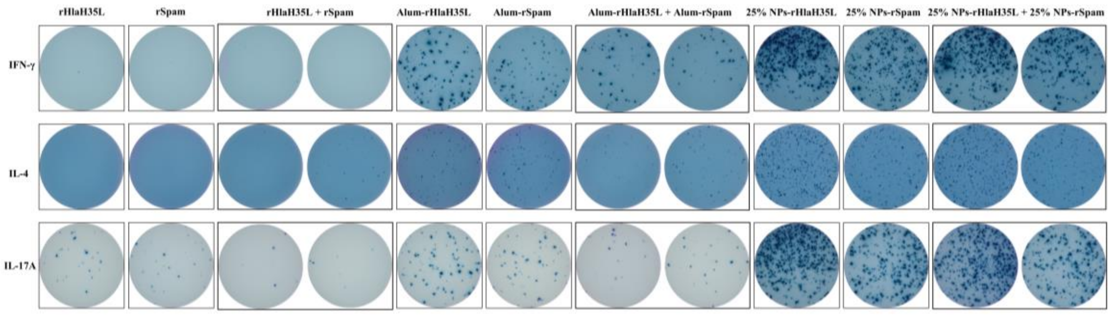

Figure S3. Representative images of ELISPOT wells are shown for cytokines produced by immunized mice splenocytes

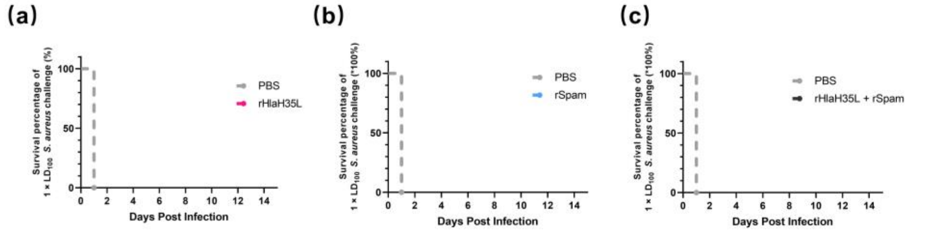

Figure S4. Survival rate comparisons on day 14 after challenge of *S. aureus* at a concentration of 2.56×10^8CFU per mouse after immunization (*n*=10)

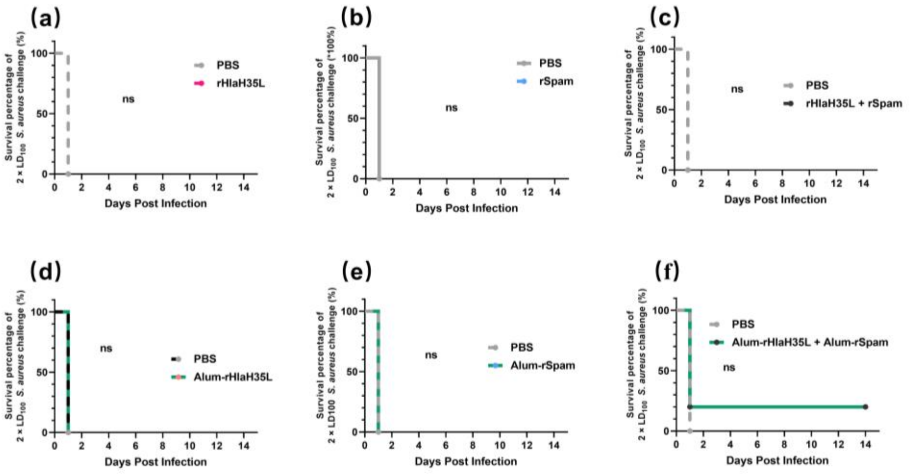

Figure S5. Survival rate comparisons on day 14 after challenge of *S. aureus* at a concentration of 5.12×10^8CFU per mouse after immunization (*n*=10)

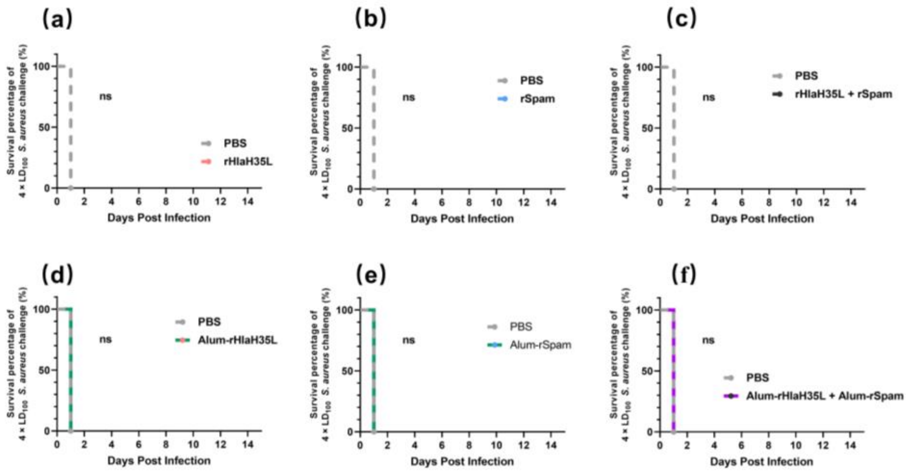

Figure S6. Survival rate comparisons on day 14 after challenge of *S. aureus* at a concentration of 1.024×10^9CFU per mouse after immunization (*n*=10)
